# Oncogenic Alternative Splicing Switches: Role in Cancer Progression and Prospects for Therapy

**DOI:** 10.1155/2013/962038

**Published:** 2013-10-27

**Authors:** Serena Bonomi, Stefania Gallo, Morena Catillo, Daniela Pignataro, Giuseppe Biamonti, Claudia Ghigna

**Affiliations:** Istituto di Genetica Molecolare, Consiglio Nazionale delle Ricerche (IGM-CNR), Via Abbiategrasso 207, 27100 Pavia, Italy

## Abstract

Alterations in the abundance or activities of alternative splicing regulators generate alternatively spliced variants that contribute to multiple aspects of tumor establishment, progression and resistance to therapeutic treatments. Notably, many cancer-associated genes are regulated through alternative splicing suggesting a significant role of this post-transcriptional regulatory mechanism in the production of oncogenes and tumor suppressors. Thus, the study of alternative splicing in cancer might provide a better understanding of the malignant transformation and identify novel pathways that are uniquely relevant to tumorigenesis. Understanding the molecular underpinnings of cancer-associated alternative splicing isoforms will not only help to explain many fundamental hallmarks of cancer, but will also offer unprecedented opportunities to improve the efficacy of anti-cancer treatments.

## 1. Introduction

Alternative splicing is the process by which splice sites in precursor messenger RNAs (pre-mRNAs) are differentially selected and paired to produce multiple mature mRNAs and protein isoforms with distinct structural and functional properties. The first example of alternative splicing was discovered almost 30 years ago, when membrane-bound and secreted antibodies were demonstrated to be encoded by the same gene [1, 2]. Now, we know that alternative splicing is a very accurate, efficient, and extraordinarily flexible process that regulates all major aspects of eukaryotic cell biology. Affecting approximately 94% of human genes [3, 4], it represents the major source of the human proteomic diversity.

Regulation of alternative splicing decisions involves the recognition of target sequences in the pre-mRNA by a number of splicing regulatory factors with antagonistic functions such as SR (serine-arginine-rich) and hnRNP (heterogeneous nuclear ribonucleoprotein) protein families [5]. Generally, SR proteins promote exon recognition by binding to exonic or intronic splicing enhancer sequences (ESEs and ISEs, resp.), while hnRNP factors typically interact with exonic or intronic splicing silencers (ESSs and ISEs) inhibiting splice sites recognition. The regulation of alternative splicing has been discussed in several excellent reviews [6–8].

Changes in alternative splicing patterns have an essential role in normal development, differentiation, and in response to physiological stimuli, but aberrant splicing generates variants that contribute to multiple aspects of tumor establishment and progression and in the resistance to therapeutic treatments [5, 9, 10]. Many cancer-associated splicing isoforms are expressed during embryonic development, but not in normal adult tissues, whereas others are entirely novel transcripts [11]. Central to the splicing oncogenic switch are changes in the expression, activity, or post-translational modification of splicing regulatory factors, such as SR and hnRNP proteins [5, 9]. Thus, modification of alternative splicing profiles contemporaneously affects multiple key aspects of cancer cell biology, including control of cell proliferation, cancer metabolism, angiogenesis, evasion from apoptosis, invasiveness, and metastasis [5, 9, 10].

Here, we discuss aberrant alternative splicing networks that contribute to the oncogenic phenotype and have a prominent role in important aspects of tumorigenesis process, including response to hypoxia and cancer cell invasion and metastasis. In addition, we also discuss important questions connected to the role of alternative splicing in cancer: what are the relevant splicing switches that are critical to malignant transformation? How the amounts/activity of the splicing regulatory factors modulate these splicing switches? What are the main functions of cancer-associated alternatively spliced variants? By illustrating specific examples, it will be clear how the production of cancer-related isoforms offers the potential to develop novel diagnostic, prognostic, and more specific anticancer therapies.

## 2. Alternative Splicing Changes of Cancer Cells in Response to Hypoxia

Through the activation of oncogenes and inactivation of tumor suppressor genes, cancer cells become able to proliferate, survive, and resist to apoptosis. Nevertheless, also microenvironmental signaling plays a crucial role in controlling cancer cell homeostasis, metabolism, growth, and differentiation [12]. The microenvironment in solid tumors is very distinct from that in normal tissues and the cross-talk between cancer and stromal cells contributes to the formation of a clinically relevant tumor and to response to antitumor therapy [13, 14]. Modifications of the microenvironment (most of these start early during tumor progression) result from metabolic alterations in cancer cells and from recruitment or activating of nontumoral cells, including blood and lymphatic endothelial cells, pericytes, carcinoma-associated fibroblasts, bone marrow-derived cells, and immune and inflammatory cells [15, 16]. In this altered microenvironment cancer cells are exposed to pro-proliferative growth factors. In addition, transformed cells often hijack the signaling circuits acting on normal cells in order to become independent from external stimulation to grow and proliferate [12, 13]. Due to deregulated cancer cell metabolism (the consequence of uncontrolled and rapid proliferation) and to an altered structure and functionality of tumor blood vessels, the tumor microenvironment is characterized by hypoxia and acidosis [15, 17, 18]. Hypoxic tumor microenvironments are now recognized as a selective pressures that promote tumor aggressiveness, inducing cancer cells to metastasize and making them refractory to radiotherapy and chemotherapy. Cells cope with hypoxia by activating transcription factor HIF-1 (hypoxia-inducible factor-1), a heterodimer formed by the constitutively expressed *β* subunit (HIF-1*β*) and the inducible *α* subunit (HIF-1*α*) [19]. The regulation of HIF-1 activity is mainly at the protein level [19]. Under non-hypoxic conditions, the HIF-1*α* subunit is rapidly ubiquitinylated and degraded by the pVHL (von Hippel-Lindau tumor suppressor protein) and the proteasome, thus preventing the dimerization with HIF-1*β* [19]. Under hypoxia, HIF-1*α* degradation is suppressed and its level increases rapidly. The HIF-1*α*/HIF-1*β* heterodimer translocates to the nucleus where it binds to hypoxia response elements (HRE) in the promoters of target genes. Through the activation of more than 150 genes, HIF-1 affects important biological processes such as angiogenesis, glucose metabolism, cell proliferation, survival, apoptosis, and invasion/metastasis [19]. Target genes include several enzymes involved in glycolysis (*Glut-1* and *PDK-1*), angiogenesis, iron metabolism (*transferrin*), cell adhesion molecules (*MIC2*), *fibronectin* and matrix metalloproteinase (*MMP-2*), extracellular matrix (ECM) modifying enzymes such as *lysyl oxidase*, and pluripotency factors including *OCT4*, *NANOG*, and *SOX2* [19–21]. 

One of the primary targets of HIF-1 is VEGFA (Vascular Endothelial Growth Factor A), a cytokine that promotes blood vessel growth and stimulates angiogenesis [22]. VEGF, secreted by hypoxic cancer cells, diffuses through the tumor mass to neighbouring host normal vessels, where, upon binding to its receptor VEGFR2, it stimulates endothelial cells to proliferate, migrate toward the tumor, and form new capillaries [23]. Even though the angiogenesis process does not initiate malignancy, it promotes tumor growth by allowing oxygen and nutrients to reach proliferating cancer cells [23]. Alternative splicing occurs extensively within *VEGF* pre-mRNA, generating various isoforms with different C-terminal domains and distinct affinity for its receptors and with a non-redundant role in angiogenesis [24, 25]. VEGF isoforms are classified in two families, called VEGF_xxx_ (pro-angiogenic) and VEGF_xxx_b (anti-angiogenic), where xxx denotes the position of the amino acid residue in the mature protein. Pro- or anti-angiogenic isoforms are generated through alternative splicing of the mutually exclusive terminal exons 8a and 8b. Selection of the proximal 5′ splice site (PSS) in exon 8a generates VEGF_xxx_ whereas the distal 5′ splice site (DSS) in exon 8b results in VEGF_xxx_b isoforms. The two types of isoforms bind VEGFR2 (Vascular Endothelial Growth Factor Receptor 2) with equal affinity. However, VEGF_xxx_ activates the downstream signaling pathways and induces angiogenesis, while VEGF_xxx_b blocks this process [26]. Given these characteristics, it is not surprising that VEGF_xxx_b is preferentially expressed in normal tissues and it is downregulated in cancer [25]. The choice between PSS or DSS depends upon the activity of the splicing factors of the SR family SRSF1 and SRSF6: SRSF6 preferentially selects the DSS and promotes the production of anti-angiogenic VEGF isoforms, whereas SRSF1 mainly activates the proximal PSS [27]. Thus, the altered expression of SRSF1 and SRSF6 in many types of tumors could impact malignant transformation by ensuring the proper balance of pro- and anti-angiogenic isoforms in cancer cells [28, 29]. Importantly, microenviromental factors can influence alternative splicing of *VEGF*. For example, treatment with TGF-*β*1 increases the anti-angiogenic VEGF_xxx_b isoforms via p38 MAPK signaling [27]. Conversely, Insulin-like Growth Factor 1 (IGF-1) decreases the expression of VEGF_xxx_b isoforms in retinal pigmented epithelial cells through PKC and SRPK1 [30]. SRPK1 is a protein kinase that specifically phosphorylates proteins containing SR domains and is upregulated in various types of cancer, like pancreatic, breast, and colon carcinoma [31]. 

Recently, a genome-wide analysis of hypoxia-induced alternative splicing changes in endothelial cells has identified target genes implicated in angiogenesis-mediated cytoskeleton remodeling (*cask*, *itsn1*, *larp6*, *sptan1*, *tpm1*, and *robo1*), in the synthesis of membrane anchors (*pign*) and in the regulation of gene expression (*cugbp1* and *max*) [32]. These changes are likely part of the adaptation of endothelial cells to stressing conditions. The impact of hypoxia on post-transcriptional events is proven also by *LAMA3-A (Laminin alpha 3)*, a splicing variant of the *LAMA3* gene induced by reduction of oxygen supply which promotes cell invasion and is associated with a poor prognosis in head and neck cancer [33, 34].

As mentioned before, HIF is a heterodimer that acts as a dominant regulator of adaptive cellular responses to hypoxia. There are three principal isoforms of the HIF-*α* subunit encoded by distinct genes: *HIF-1*α**, *HIF-2*α** (*EPAS1*), and *HIF-3*α**. *HIF-3*α** gene consists of 19 exons and it is subjected to extensive alternative splicing leading to the production of at least six isoforms [35]. HIF-3**α**4 is one of the HIF-3**α** splicing variants whose functions have recently been linked the development of hypervascular malignant meningiomas [36]. Indeed, HIF-3**α**4 directly binds to HIF-1**α** and suppresses HRE dependent transcription of *VEGF*. Importantly, HIF-3**α**4 is able to inhibit proliferation and invasion, to reduce neovascularization and glucose metabolism in hypervascular meningiomas [36].

Another gene induced by hypoxia is *Cyr61 *(cysteine rich 61) encoding for a secrete protein that functions as a proangiogenic factor promoting adhesion, migration, and survival of vascular endothelial cells [37]. Cyr61 is supposed to be a promoter of tumor progression since its high expression levels were detected in various cancer types [38, 39]. Notably, in addition to transcriptional level, the expression of Cyr61 can be also regulated through an alternative splicing event that stimulates the retention of intron 3 in *Cyr61 *mRNA leading to the production of a nonsense-mediated decay (NMD) sensible transcript [40]. Importantly, this alternative splicing event coupled with NMD pathway (called AS-NMD) was reported to be altered in breast cancer and associated with a shift from an intron 3 retained transcript (IR) toward an intron 3 spliced mRNA (IS) encoding for the biological active Cyr61 protein. Moreover, in several breast cancer cell lines under hypoxic conditions AS-NMD of *Cyr61* was reported to enhance the expression of the IS transcript, suggesting that hypoxia-mediated alternative splicing changes could be a central mechanism regulating the Cyr61 expression and its tumor-promoting activity [40].

The CD44 glycoprotein provides another attractive example of hypoxia-induced alternative splicing changes. CD44 is a transmembrane molecule able to bind several ligands, including important components of ECM, such as hyaluronic acid, collagen, fibronectin, laminin, and matrix metalloproteinases (MMPs), and involved in cell-cell and cell-matrix interactions, migration, and invasion [41]. CD44 pre-mRNA consists of 20 exons, 10 of which (v1–v10) undergo alternative splicing events, thus generating multiple CD44 isoforms with different molecular sizes and with diverse extracellular domains [41, 42]. The predominant CD44 isoform, the low-molecular-weight CD44s (90 kDa standard form), is expressed by several tissues, including hematopoietic, fibroblast, and glial cells, whereas high-molecular-weight CD44v variants (140–230 kDa) are restricted to epithelial cells and abundant in epithelial-type carcinomas [41, 42]. The physiological/pathological functions of most CD44v variants remain still poorly understood. However, it has been observed that some variants are over-expressed in various tumors and implicated in cancer cell invasion and metastasis [41, 43, 44]. In particular, CD44v6 and CD44v8 variants are associated with tumor progression and poor diagnosis in several types of carcinoma including breast and colorectal cancers [45–47]. Interestingly, HIF-1**α** is able to increase the expression of the *CD44* mRNA and to upregulate CD44 variants containing exons v6 and v7/8 [48]. In line with this, Krishnamachary and collaborators have reported that hypoxic regions of breast cancer specimens contain cells with elevated expression of CD44 [48]. Additional studies are necessary to identify the molecular mechanisms and signaling pathways regulating *CD44* alternative splicing in response to hypoxia.

Hypoxia-induced alternative splicing changes were also recently investigated in the study of Hirschfeld and colleagues [49]. The *YT521* (YTH domain containing 1) is a ubiquitously expressed nuclear splicing factor containing a novel RNA-binding domain (YTH domain) necessary for YT521 to directly influence splice site selection. Importantly, low YT521 expression was associated with clinical outcome in patients with type I endometrial cancer, suggesting its potential role as a tumor suppressor [50]. Furthermore, YT521 alternative splicing targets are well-known cancer-associated genes such as *BRCA2*, *ESR1*, *MDM2*, *VEGF*, and *CD44* [49, 51–55]. Similar to other proteins involved in mRNA processing, the expression of YT521 is regulated by alternative splicing. Interestingly, it was reported that under hypoxic conditions the alternative splicing of *YT521 *pre-mRNA containing exons 8 and 9 generates two transcripts that are subjected to degradation through NMD, suggesting that these AS-NMD events of YT521 could simultaneously affect the processing of several cancer-associated genes.

Further functional investigations on hypoxia and its impact in regard to alternative splicing of target genes may contribute to better understanding of a key regulatory epiphenomenon in tumor growth involved in the development of an aggressive cancer phenotype.

## 3. Invasion and Metastases

The term metastasization is currently used to indicate the ability of tumor cells to invade adjacent tissues and disseminate toward distant organs [56]. This is a multistep process that involves (i) local invasion of tumor cells through the basement membrane and endothelial walls into the host stroma, (ii) intravasation within the blood and the lymphatic circulatory systems; (iii) extravasation into distant tissues, and (iv) proliferation of tumor cells allowing growth and efficient metastatic colonization [56].

As observed for other hallmarks of cancer, gene expression programs implicated in the metastatic process are the same that participate in embryonic development, morphogenesis, and wound healing [57, 58]. One of these programs that physiologically pertain to embryogenesis is the epithelial-to-mesenchymal transition (EMT). The EMT process involves dedifferentiation steps in which cells lose their epithelial phenotype to acquire mesenchymal traits [58, 59]. The epithelial cell layer consists of polarized cells with cohesive cell-cell junctions. Through EMT cells undergo an extensive reorganization of the cytoskeletal architecture with the loss of intercellular junctions and cell polarity and the acquisition of an elongated, fibroblast-like shape. During tumor progression, EMT is one of the major routes through which cancer cells acquire invasive capabilities and spread throughout the body as single cells [58, 59]. Importantly, tumor EMT is a transient process that occurs in a subset of cells at the invasive front of the metastasizing primary carcinoma and is reversed at the final metastatic sites, where cells undergo the reverse process, namely, the mesenchymal-to-epithelial transition (MET) [58, 59]. This plasticity and the redifferentiation of metastatic cells to an epithelial identity help in organ colonization, ensuring metastasis formation. Moreover, it clearly indicates that EMT is not driven by stable genetic mutations but by activation of gene expression programs in response to external cues in the tumor microenvironment. 

Several relevant players involved in EMT/MET cycles have been identified including transcription factors, growth factors, cytokines and chemokines, pro-angiogenic factors, cell adhesion molecules, modifiers of cytoskeletal organization, and extracellular matrix-remodeling enzymes [60]. The prevailing models propose that these regulators act largely at transcriptional level. Indeed, several pathways activate a network of transcription factors that promote the expression of mesenchymal markers, such as N-cadherin and vimentin, while inhibiting E-cadherin production, not only a key component of adherent junctions but also a tumor suppressor frequently repressed, mutated or degraded during tumor transformation [58, 59]. However, in the last few years, several studies have highlighted additional layers of EMT control, including epigenetic reprogramming, small noncoding RNAs, translational and post-translational regulations, and alternative splicing changes [10, 57, 61, 62]. In particular, an increasing body of evidence indicates that splicing regulation alone can drive critical aspects of EMT-associated phenotypic changes [10]. Below, we discuss some interesting examples of specific alternative splicing events that are implicated not just in the EMT/MET program but also in the different stages of the metastatic cascade.

### 3.1. Ron

Alternative splicing of the *Ron* proto-oncogene (also known as MST1R) has provided the first example of an alternative splicing variant linked to the activation of tumor EMT. *Ron* encodes for a tyrosine kinase receptor involved in control of cell dissociation, migration, and matrix invasion. The activity of the Ron receptor is regulated through alternative splicing. In particular, a constitutively active isoform (called ΔRon), which confers increased motility to expressing cells and accumulates during tumor progression of epithelial cancers [63, 64], is generated through skipping of exon 11. The oncoprotein SRSF1 deeply impacts cell physiology since its upregulation stimulates skipping of exon 11, thus promoting the production of ΔRon isoform that in turn triggers activation of the EMT program increasing the invasive properties of the cells [64]. Interestingly, SRSF1 expression levels are dynamically controlled in epithelial and mesenchymal cells through an AS-NMD event [65]. AS-NMD of *SRSF1*, which involves an intron in the 3′UTR region of the gene, decreases mRNA stability and SRSF1 protein levels and, notably, it is altered in colon cancer. This scenario is further complicated by the involvement of another splicing regulator, Sam68. Sam68, the 68 kD Src-associated protein in mitosis, is a member of the STAR (signal transduction and activation of RNA) family of RNA-binding proteins [66]. It contains a single KH-type RNA-binding domain and several protein-protein interaction motifs including potential binding sites for SH2, SH3, and WW domains, which are characteristic of signaling transducers [67]. Sam68 has been recognized as a substrate of several kinases, such as members of the Src family, ERK1/2 and BRK (Breast Tumor Kinase) [65, 68–70]. As such, Sam68 is the first identified “hub factor” able to communicate extracellular stimuli to specific RNA splicing decisions. In particular, directed by ERK1/2 signaling, Sam68 controls AS-NMD of *SRSF1* transcript, thus modulating its protein level. Notably, epithelial cell-derived soluble factors are able to inhibit ERK1/2 signaling thereby repressing Sam68 phosphorylation, which increases the production of the NMD-sensitive transcript of *SRSF1*.

### 3.2. Rac1

The matrix metalloproteinases (MMPs) are the most important family of proteinases of the tumor microenvironment that degrade structural components of the extracellular matrix (ECM), thus regulating proliferation, cell-cell adhesion, angiogenesis, invasion, and metastases [71]. In line with this, MMPs are upregulated in almost all types of human cancer and associated with poor survival [71]. Notably, over-expression of MMP-3 in mammary and lung epithelial cells triggers a cascade of events that determine activation of EMT process [72, 73]. Notably, these events, which induce tumorigenesis process in transgenic mice, are dependent on the expression of a constitutively active alternatively spliced isoform of the *Rac1* gene, encoding for a small GTPase of the mammalian Rho family involved in actin cytoskeleton organization, cell growth, cell-cell adhesion, and migration [74]. This splice variant (known as Rac1b) is produced by inclusion of the exon 3b that contains the encoding region for a 19-amino acid domain involved in the interaction with regulator and effector molecules [75]. Since Rac1b shows increased expression during progression of several cancer types [72, 75, 76], it is tempting to speculate that the exon 3b (or the 19-amino acid insertion) could offer the opportunity for a selective targeting to develop anticancer therapies that block EMT-associated progression towards advanced tumor stages.

### 3.3. KLF6

The complexity of networks regulating the EMT program makes it very difficult to identify early inducers and a unifying molecular basis for this transition. Many transcription factors have been extensively studied for their involvement in activation of EMT. Among these are Slug (referred also to as SNAI2) and Twist, two repressors of *E-cadherin* promoter activity [57]. Recent studies have revealed important roles for specific alternatively spliced variants of upstream regulators of Slug and Twist activity. This is the case of oncogenic splice variant 1 of *KLF6* (KLF6-SV1), a tumor suppressor gene belonging to the Krüppel-like family of transcription factors, able to act as functional driver of the entire metastatic cascade through Twist induction [77, 78]. Notably, KLF6-SV1 antagonizes the tumor suppressive activity of the full-length KLF6 protein and sustains tumor growth and dissemination in ovarian and prostate cancer models [79–81]. Interestingly, increased expression of KLF6-SV1 occurs in many tumors and is associated with poor prognosis in prostate, lung, and ovarian cancers [82]. KLF6-SV1 has been recently shown to induce EMT and to drive aggressive multiorgan metastasis formation in both subcutaneous and orthotopic mouse models of breast cancer [78]. In this process, it acts through a Twist-dependent mechanism and Twist downregulation reverts the phenotype of KLF6-SV1 over-expressing cells, restoring the expression of epithelial markers [78]. Finally, high levels of KLF6-SV1 were found associated with increase in EMT markers in a large cohort of primary breast cancer patients [78].

### 3.4. FAM3B

*FAM3B* is a member of the novel *FAM3* family of cytokine-like genes, predicted to produce at least 7 alternatively spliced isoforms [83, 84]. The most studied variant is the secreted form PANDER, so called for its robust expression and activity in the pancreatic cells [85, 86]. In addition, lower PANDER levels have been observed in human gastric cancers in respect to the corresponding normal tissues [87]. Recently, Li and colleagues have identified FAM3B-258, a 258-amino acids non-secretory protein, as a novel splicing variant of FAM3B up-regulated in colon adenocarcinoma cell lines as well as in human colorectal tumors [84]. FAM3B-258 is able to induce changes typical of an activated EMT program, stimulating cell migration and invasion *in vitro* and promoting metastases formation in nude mice. The oncogenic abilities of FAM3B-258 require a Slug-mediated transcriptional repression of *E-cadherin* and *JAM* (Junctional Adhesion Molecule). Knock-down of *Slug* in FAM3B-258 over-expressing cells restores higher levels of E-cadherin and JAM and prevents the FAM3B-258-dependent cell invasion [84]. Whether Slug is a direct target of FAM3B-258 or whether other signaling effectors are involved remains to be investigated.

### 3.5. Cortactin

To become migratory and invasive, cells must extend plasma membrane protrusions (such as lamellipodia and filopodia) forward and overcome the epithelial basement layer as a first barrier [56, 88]. The formation of invadopodia has been recently characterized as another important step of the EMT program [89–91]. Invadopodia are enriched with a variety of proteins, including actin and actin regulatory proteins [92, 93]. The filamentous actin (F-actin) binding protein cortactin is one of the regulators of actin polymerization/branching involved in invadopodia assembly and maturation [92, 94]. Three splicing products of cortactin have been characterized until now [95]. Unlike the full-length protein (FL), the SV1 and SV2 variants lack, respectively, one and two of the six “cortactin repeats” that mediate the interaction with F-actin [95]. As a consequence of their reduced capabilities in F-acting binding and polymerization, over-expression of SV1- or SV2-cortactin shows reduced cell migration when compared with FL-cortactin over-expressing cells [95]. One of the factors that control alternative splicing of *cortactin* transcripts is SPF45 [96]. Initially characterized as a component of the spliceosome, SPF45 has been also implicated in the regulation of *Fas* and *fibronectin* alternative splicing [97, 98]. In their interesting article, Liu and coworkers have demonstrated that SPF45 mediates cell migration and invasion in ovarian cancer cells by promoting the formation of the FL-cortactin isoform. SPF45 activity appears to be controlled via phosphorylation by Clk1 (Cdc2-like kinase). Finally, SPF45 over-expression correlates also with increased cortactin phosphorylation by ERK, which enhances cortactin-mediated actin polymerization [96, 99].

### 3.6. MENA

As for invadopodia, filopodia nucleation and extension require actin cytoskeleton remodeling and involve several regulators of actin dynamics, such as the Ena/VASP protein family that in mammals includes three members: MENA (also called ENAH), VASP, and EVL [88]. Up-regulation of MENA has been detected in several human cancers, including breast cancer and melanoma and correlates with invasiveness of breast tumors [100, 101]. *MENA* pre-mRNA is alternatively spliced to generate different isoforms, expressed in a tissue-specific manner [102]. Importantly, the alternative splicing profile of *MENA* in invasive tumor cells is different from non-migratory resident cancer cells. Non-invasive tumor cells as well as poorly invasive breast cancer cells with epithelial morphology express MENA^11a^, the epithelial-associated variant generated from inclusion of exon 11a [102, 103]. This exon is inserted within the EVH2 domain, very close to the F-actin binding motif and the tetramerization site. This insertion has been predicted to affect the ability of MENA tetramers to interact with F-actin and thus to drive filopodia and lamellipodia maturation [103]. In agreement with this, the increment of MENA^11a^ reduces both the number and the length of filopodia in a 3D culture assay [104]. On the contrary, invasive cancer cells lack MENA^11a^ and express MENA^INV^, an isoform containing an additional exon, also referred to as exon +++ [103, 105]. The relative abundance of MENA^INV^ and MENA^11a^ seems to be important to regulate key stages of the metastatic cascade in breast cancer cells. Thus, high levels of MENA^INV^ enhance coordinated motility, transendothelial migration, and intravasation of tumor cells, promoting spontaneous lung metastases in a murine model of breast cancer [105, 106]. In contrast, increased expression of MENA^11a^ correlates with decreased invasion, intravasation, and dissemination of cancer cells [106]. Recently, Di Modugno and coworkers have identified another splice variant of human *MENA* lacking exon 6 and called hMENAΔv6 [104]. Contrary to MENA^11a^, hMENAΔv6 is expressed selectively in invasive cancer cells with mesenchymal phenotype and is able to enhance invasiveness of breast cancer cells but only in the absence of MENA^11a^ [104]. This evidence suggests that MENA^11a^ behaves as dominant anti-invasive player, making alternative splicing of this gene a potential target for anticancer therapies. Furthermore, *MENA* splicing occurs also in primary breast tumors and in particular MENA^11a^-negative tumors display lower level of E-cadherin when compared to MENA^11a^-positive samples [104], supporting the anti-migratory functions of this splicing isoform [104].

Splicing of *MENA* exon 11a is regulated by the expression levels of epithelial-specific alternative splicing factors ESRP1 and 2 (Epithelial Splicing Regulatory Proteins 1 and 2), two important regulators of the mesenchymal and epithelial splicing signatures [107]. In particular, ESRP proteins, by promoting inclusion of exon 11a and the production of MENA^11a^ isoform, cause a drastic reorganization of actin cytoskeleton as well as cell morphology and a reduction of invasive properties [107]. In addition to MENA, ESRPs control the alternative splicing of several genes involved in different aspects of the metastatic cascade, such as cell-cell and cell-matrix adhesion, actin cytoskeleton organization, cell polarity, and migration [108, 109]. In line with this, modification of ESRPs expression levels results in alternative splicing changes of CD44 and p120-Catenin, a protein found at cell-cell junctions and also involved in signal transduction [108, 109].

### 3.7. L1CAM

The penetration of migrating cancer cells into tissue barriers, including the basement membrane, is supported by the proteolytic degradation of the extracellular matrix (ECM) through the activity of secreted enzymes, such as matrix metalloproteinases MMP-2 and MMP-9 [110]. The expression and activity of MMPs are regulated through several signals, mainly induced by growth factors and chemokines, as well as through integrin and extracellular matrix-mediated signals [110]. Recently, the alternative splicing of the cell adhesion molecule *L1* (*L1CAM*) has been found to control the invasive capabilities of tumor cells by regulating MMP-2 and MMP-9 expression and activity [111]. More specifically, although initially the splicing variant considered as cancer-associated was SV-L1CAM (lacking of exons 2 and 27), only the full-length FL-L1CAM has been found up-regulated upon exposure of tumor cells to the pro-metastatic factors TGF-*β* and HGF. Importantly, the over-expression of FL-L1CAM but not of the SV isoform is able to induce metastasis formation in mice [111].

### 3.8. SVEP1

Invasion and colonization of a secondary organ by disseminating cancer cells are influenced by the microenvironment and the cross-talk between cancer populations and cells in the niche of the receiving tissue [56, 112]. The cell adhesion molecule SVEP1 has been recently involved in the interactive network that affects breast cancer cells homing to bone niches [113]. SVEP1 expression is stimulated by TNF*α*, a pro-inflammatory cytokine able to affect adhesion and migration, and to induce EMT [114, 115]. Recently, Glait-Santar and colleagues have investigated the alternative splicing of SVEP1 transcripts in a co-culture model of pre-osteoblastic MDA-15 and mammary adenocarcinoma DA3 cells, which mimic the molecular interactions in the bone niche after invasion of breast carcinoma cells [116]. Similar to what observed after TNF*α* treatment, several splicing isoforms of SVEP1, such as the full-length isoform a and isoform e, are up-regulated in both cell lines upon co-culture conditions. In parallel embryonic variants g and f are silenced in adenocarcinoma DA3 cells, whereas no effect is observed in pre-osteoblastoma cells [116]. The same authors also observed that the ratio between splicing isoforms of SVEP1 is perturbed after treatment with epigenetic drugs such as DNA demethylating or histone deacetylase inhibitors, supporting a link between the epigenetic organization and splicing of *SVEP1* pre-mRNA. However, further studies are needed to establish the pathological role of the different SVEP1 isoforms in the metastatic process.

### 3.9. CD44

Several microenvironmental stressors, including nutrient deficiency, low pH, mediators of inflammatory responses, and reactive oxygen species (ROS), can affect successful colonization by disseminating cells at the final metastatic tissues [117]. Disseminating cancer cells can take advantage of antioxidant systems to counteract the exposure to oxidative stress. The synthesis of reduced glutathione (GSH), a reducing thiol peptide, protects cancer cells against ROS-mediated damage and confers resistance to anticancer therapies [118, 119]. A rate-limiting factor for GSH synthesis is the availability of cysteine and the cystine transporter system xc- (composed by two subunits, xCT and CD98hc) is essential in the GSH antioxidant mechanism [119, 120]. Recently, a link between the GSH-dependent evasion from oxidative stress and alternative splicing of *CD44* has been identified [121, 122]. As mentioned before, CD44 has an important role in cell-cell and cell-matrix interactions, migration, and invasion [41]. Through alternative splicing, *CD44* pre-mRNA generates multiple CD44 high-molecular-weight isoforms with different extracellular domains [41, 42]. Intriguingly, CD44v8-10 has been demonstrated to interact with the cystine transporter xCT, increasing the levels of GSH and, as a consequence, the ability of cancer cells to avoid ROS damage [122]. In line with this, CD44v-positive 4T1 mouse breast cancer cells display high levels of GSH and xCT and enhanced ROS defense compared to CD44v-negative 4T1 cells [123]. Thus, CD44v-positive 4T1 are able to establish lung metastatic lesions in mouse models with an incidence higher than CD44v-negative cells [123]. Interestingly, down-regulation of the splicing regulator ESRP1 in metastatic 4T1 cells shifts the splicing pattern toward the production of CD44s and results in suppression of lung metastasis [123]. On the contrary, forced expression of CD44v8-10 in ESRP1-depleted cells is sufficient to restore high content of GSH and lung colonization potential [123].

## 4. Diagnostic, Prognostic, and Anticancer Therapy Potentials of Alternative Splicing

Cancer chemotherapy relies on the expectation that anticancer drugs will preferentially kill rapidly dividing tumor cells, rather than normal cells. Unfortunately, most pharmacological approaches for the treatment of solid tumors suffer from poor selectivity, which limits the overall dose of drug that can be administered because of unacceptable toxicities to normal tissues. 

As shown in previous sections, alternative splicing variants of many cancer-related genes can directly contribute to the oncogenic phenotype and to the acquisition of resistance to therapeutic treatments [5, 9, 10]. Alternative splicing isoforms selectively expressed by tumors and not by normal tissues may represent suitable targets for new therapeutical approaches [11]. In this section, we discuss some significant examples to illustrate how cancer-specific splicing events can be a powerful source of new diagnostic, prognostic, and therapeutic tools.

Several highly sensitive methods allowed the identification of cancer-specific splicing isoforms [124–128]. For example, the splicing profile of *ABCC1*, *Mdm2,* and *fibronectin* transcripts has been used to distinguish normal ovary from epithelial ovarian cancer [127], whereas altered splicing of *MED24*, *MFI2*, *SRRT*, *CD44*, and *CLK1* has been associated with metastatic phenotype in breast cancer and poor prognosis in patients [128]. Notably, splicing of *hMENA* may improve the early diagnosis of breast cancer and clinical decision [104], whereas the balance between splicing variants of *KLF6* and *caspase-9* genes could be useful to predict the susceptibility of cancer cells to chemotherapy [129, 130].

Along this line, a SpliceDisease database (http://cmbi.bjmu.edu.cn/sdisease), which provides information for relationships among gene mutations, splicing defects, and disease, has been recently developed [131]. A list of cancer-specific alternative splicing isoforms is shown in Table 1.

Cancer-specific splice variants may not only serve as diagnostic and prognostic tumor biomarkers but also provide potential targets for the development of new therapeutic strategies. Promising avenues towards the development of more selective anticancer drugs are (i) antibodies against tumor-associated markers, (ii) small molecules targeting the spliceosome or *trans*-acting splicing regulatory factors, and (iii) antisense oligonucleotides that prevent the production of specific aberrant alternative splicing variants. 

### 4.1. Monoclonal Antibodies Targeting Splicing Isoforms

**Figure 1:**
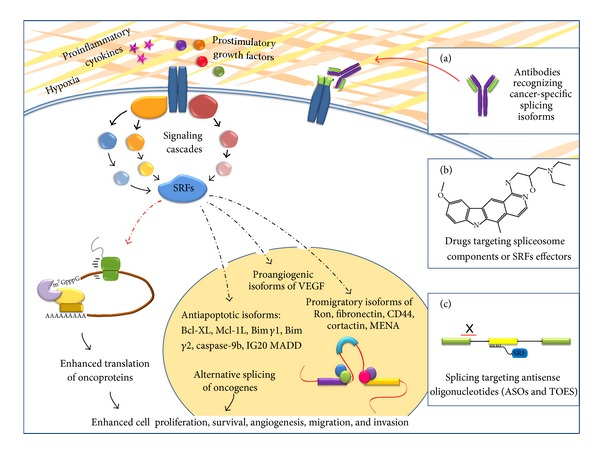
SRFs (splicing regulatory factors) at the cross-road between oncogenic signaling pathways and targets for anticancer treatments. During tumorigenesis, cancer cells are exposed to stressing conditions such as hypoxia and acidosis. In this altered tumor microenvironment, growth factors and cytokines, provided by either cancer or non-tumoral cells, activate signaling cascades affecting both the activity and/or the expression levels of splicing regulatory factors (SRFs). In the cytoplasm, SRSFs can enhance the translation of oncogenic variants involved in key aspects of cancer cell biology. In the nucleus, SRFs are mainly involved in the regulation of alternative splicing of pre-mRNAs relevant to cancer progression mechanisms, namely, pro-liferation, angiogenesis, survival, invasion, and metastasis. Alternative splicing variants of cancer-related genes represent powerful targets for new therapeutic approaches. (a) Alternative splicing can generate unique epitopes in cell surface proteins that can be targeted by monoclonal antibodies (mAbs), able to lead to down-regulation or neutralization of the specific isoforms. Moreover, mAbs can be also used to selectively deliver bioactive molecules to cancer cells without affecting normal tissues. (b) Small molecules, by interfering with the spliceosome assembly or with the phosphorylation status of SRFs (i.e., SR proteins), can in turn affect the balance of alternative splicing products, preventing the generation of cancer-associated variants. (c) Standard ASOs (antisense oligonucleotides) block the interaction between the splicing machinery and the cognate splicing sequences (splice sites, enhancer or silencer elements), whereas TOES (targeted oligonucleotide enhancers of splicing) oligonucleotides contain a “tail” of ESE sequences to recruit SRFs on a specific alternative exon. By inhibiting or activating specific splicing events, TOES can be used to shift the ratio between biologically functional splice variants toward the production of non-pathological isoforms.

Alternative splicing in cancer can generate unique epitopes in the extracellular domain of cell membrane proteins. Indeed, many receptors involved in cell-cell and cell-matrix interactions undergo alternative splicing and specific splicing isoforms are associated with human malignancies [152]. These novel, or embryo-restricted, epitopes seem to be ideally suited for tumor-targeting strategies consisting in the delivery of bioactive compounds, for example, monoclonal antibodies (mAbs). Binding of mAbs to tumor-associated biomarkers can determine down-regulation or inhibit the function of the target (Figure 1(a)). Moreover, when conjugated with radioemitters or chemotherapics, the mAbs can efficiently ensure *in situ* delivery of the bioactive molecule to cancer cells, sparing normal tissues.

A very well studied target for the mAbs-mediated therapy is the Epidermal Growth Factor Receptor (EGFR), which is over-expressed in several tumors [153]. Two antibodies directed against the EGFR, cetuximab (C225) and panitumumab, are currently used in therapy [154]. Unfortunately, since EGFR is expressed also in normal tissue, this therapy may have severe side effects. Interestingly, several tumor-specific splice variants of EGFR have been identified. One of this is the EGFRvIII variant, and two mAbs (Ch806 and CH12), targeting the unique extramembrane epitope of EGFRvIII, are used in clinical trial [155, 156]. Another cancer-specific EGFR isoform, de4 EGFR, is also recognized by mAb CH12 [157, 158]. Treatment with CH12, but not with cetuximab, of mice over-expressing the de4 EGFR variant significantly suppresses tumor proliferation and angiogenesis, leading to tumor apoptosis [158]. 

An important correlation between aberrant alternative splicing and tumor progression has been shown for CD44. In particular, CD44 isoforms containing the variant exon v6 and v8 (CD44v6 or CD44v8) are commonly over-expressed in epithelial tumors [159]. Unfortunately, the expression of these isoforms is not confined to cancer cells, but it occurs also in normal tissues, as skin keratinocytes [160]. Various mAbs targeting CD44v6 have been evaluated in clinical trials [152]. Anti-v6 mAbs are effective in treatment of patients with head and neck squamous cell carcinoma but they show severe skin toxicity [161, 162]. Recently, Masuko and collaborators have developed a mAb (GV5) against CD44R1, a CD44 isoform containing exons v8, v9, and v10 [163]. GV5 exhibited therapeutic effects in xenografts models, probably by inducing antibody-dependent cellular cytotoxicity, with undetectable reactivity with skin keratinocytes. However, despite these promising developments, solid tumors are frequently resistant to antibody-based therapies probably for the poor penetration of antibodies into the tumor tissue. Neo-vascularization (or angiogenesis) is needed for growth of cancer cells and for the metastatic process [22]. Tumor endothelial cells have a central role in this process because they are readily accessible to drugs via the blood circulation. Exploiting this feature, new cancer therapies have been developed to target the tumor vasculature with the aim to deprive the tumor of oxygen and nutrients and induce its regression [164]. Endothelial cells of tumor vessels express splicing isoforms of matrix proteins such as the fibronectin (FN) [165, 166]. For example, the oncofetal isoform containing the extra domain EDB of FN is exclusively expressed around newly developing tumor vasculature, whereas it is absent in adult tissue [165]. Notably, EDB-specific radiolabeled antibodies are used in clinical trial for antiangiogenic cancer treatment [167].

### 4.2. Small Molecules Targeting Splicing Components

The first drugs used to target the spliceosome machinery, FR901464 and herboxidiene, are natural compounds extracted from bacteria [168, 169]. Subsequently, synthetic analogues, with less complex structure, therefore with minor synthesis costs, but with higher stability, solubility, and activity, have been obtained [168]. All these molecules have been demonstrated to have selective toxicity and anti-cancer properties in human tumor xenografts [168]. Their molecular mechanism has been recently elucidated [170]. They bind to the splicing factor 3b (SF3b), destabilizing the U2 snRNP-pre-mRNA complex and altering the conformation of the branch site sequence. As a consequence, the correct selection of the 3′ splice acceptor site fails to occur and alternatively spliced mRNAs are generated [170]. One more promising antitumor agent characterized for its anti-proliferative activity is the biflavonoid isoginkgetin, a natural product found in a variety of plants [171]. Interestingly, isoginkgetin inhibits the transition from pre-spliceosomal complex A to complex B, probably by preventing stable recruitment of the U4/U5/U6 nuclear ribonucleoproteins [171].

SR proteins are targets of extensive phosphorylation that influences protein interactions and regulates their activity and sub-cellular localization [5] (Figure 1(b)). The benzothiazole compound TG003 has been described as a potent inhibitory of Clk1/Sty able to affect SFSR1-depending alternative splicing events [172]. Interestingly, the exposure of human colon carcinoma cells to TG003 has been described to determine accumulation of the tumor suppressor p53 [173], the most commonly mutated gene in human cancers. Activation of p53 is promoted by down-regulation of MdmX and decreased stability of Mdm2, two key repressors of p53. Interestingly, TG003 seems to contribute to their degradation rather than to change their pre-mRNA splicing. Further studies are required to elucidate the role of p53 pathway as a sensor of alterations in the splicing machinery.

As mentioned before, SRSF1 and SRSF6 control the choice between VEGF_xxx_ (angiogenic) and VEGF_xxx_b (anti-angiogenic) isoforms. Interestingly, phosphorylation of SRSF1 by SRPK1 promotes the use of the proximal splice site within exon 8 of *VEGF* pre-mRNA and thus the production of the angiogenic isoform, while phosphorylation of SRSF6 activates the distal splice site in exon 8 [30]. Along this line, inhibitors of SRPK1 and Clk functions, as SRPIN340 or TG003, have been shown to block SRSF1 activation and to inhibit angiogenesis process both *in vitro* and *in vivo* [30, 174].

Indole derivatives represent a new class of strong splicing inhibitors able to interact with SR proteins and prevent their phosphorylation [175]. Some of them show anti-proliferative activity, with an acceptable toxicity [175]. In our recent study, we have used indole derivatives to modulate the splicing event that generates the cancer-associated ΔRon variant [176]. Binding of SRSF1 to an ESE sequence within exon 12 leads to skipping of exon 11 and to the production of the constitutively active ΔRon isoform [64]. Interestingly, three indole derivatives can reverse aberrant *ΔRon* splicing. Among these, only IDC92 is able to revert the invasive phenotype of cancer cells without affecting the splicing profile of other SRSF1 targets, suggesting that this small molecule is suitable for further *in vivo* studies [176].

Because of their lack of specificity in modulating pre-mRNA splicing, these compounds are expected to cause deleterious undesired events in normal cells as well. Surprisingly, however, most of them have been found to possess selective tumor cytotoxicity. One hypothesis is that tumor cells respond differently from normal cells to changes in mRNA splicing. Alternatively, transformed cells may differ from normal counterparts for the expression of modified version of tumor suppressors originated by aberrant splicing and drug treatment may reverse this defect [168]. However, molecular mechanisms underlying this specificity toward cancerous cells remain elusive and additional studies are necessary to characterize the effects of these drugs in both tumor and normal cells. 

Several works have recently demonstrated that also inhibitors of oncogenic pathway components can indirectly target splicing reactions. For example, treatment of melanoma cells, harbouring B-Raf (V600E) mutation with B-RAF inhibitors, determines over-expression of SRSF6 that in turn regulates alternative splicing of the *Bim* gene, a member of the Bcl-2 family, promoting the production of the proapoptotic short isoform Bim_S_ [177, 178].

### 4.3. Oligonucleotides-Mediated Therapies

Antisense oligonucleotides (ASOs) are short oligonucleotides, usually 15–25 bases in length, that are designed to anneal to a specific target region on a pre-mRNA molecule, thus interfering with the splicing reaction (Figure 1(c)). ASO targeting an exon-intron junction may sterically block the access to the splicing machinery, redirecting splicing reaction to an adjacent splicing site. Alternatively, ASO can bind to splicing enhancer or silencer elements, masking the sequence to *trans*-acting regulatory factors and determining inclusion or skipping of specific exons. 

The latest generations of ASOs contain chemical modifications and appear more stable compared to conventional oligonucleotides. All ASOs share the following characteristics: (i) they bind tightly to RNA through Watson-Crick base-pairs; (ii) they are specific for RNA molecule, and (iii) they do not alter the genomic sequence. In addition, other features make them appreciable therapeutic tools. Indeed, the delivery technology, usually nanoparticle, is noninvasive, nontoxic, efficient, and very stable. 

Clinical trials have been already started that exploit ASOs for treatments of human genetic disorders [179]. Even though the use of these molecules in anti-cancer therapy is still at early stages [180], several recent works report therapeutically relevant and encouraging results. Below, we describe several studies, some of them performed on xenograft models of human tumors, while the more recent preliminary results on cancer cell lines are listed in Table 2.

The first demonstration of *in vivo* anti-tumor efficacy of ASOs was reported by Bauman and colleagues [181]. The authors challenged a modified ASO, targeting the downstream 5′ alternative splice site of exon 2 in *Bcl-X* pre-mRNA (Bcl-X ASO), in a mouse model of metastatic melanoma, an aggressive malignancy that shows poor prognosis when associated with increased expression Bcl-X_L_ splice variant [182]. The oligo efficiently redirected splicing machinery to the upstream 5′ splice site, decreasing the anti-apoptotic Bcl-X_L_ isoform, while increasing the pro-apoptotic Bcl-X_S_ variant [181]. Importantly, the administration of the oligo coupled to nanoparticles produced a significant reduction of tumor burden in rapidly growing and highly tumorigenic lung metastases [181].

Another example is the exploitation of ASOs to efficiently modify splicing of *STAT3*, another gene involved in apoptosis. The usage of an alternative acceptor site within exon 23 of *STAT3* pre-mRNA leads to the production of the truncated STAT3*β* isoform that promotes apoptosis and cell-cycle arrest [183]. Interestingly, by using a modified ASO, targeting a splicing enhancer element that regulates alternative splicing of exon 23, it was possible to promote a shift from STAT3*α* to STAT3*β* leading to tumor regression in a xenograft model of cancer [184]. 

Recently Cartegni's group showed that the antagonism/association between intronic polyadenylation and pre-mRNA splicing can produce truncated soluble receptor tyrosine kinases (RTKs). These isoforms can act as dominant-negative regulators since they are deficient of the anchoring transmembrane and the intracellular kinase domains [185]. Notably, these secreted “decoy receptors” are able to inactivate the associated tumorigenic signaling pathways as a consequence of their ability to titrating the ligand or by blocking the wild-type receptors in non-functional heterodimers [185]. Interestingly, in the case of the Vascular Endothelial Growth Factor Receptor 2 (VEGFR2/KDR), the key molecule involved in the control of the VEGF signaling, morpholino antisense oligonucleotides (ASOs extremely stable within biological systems because they are resistant to a wide range of nucleases) were used to induce the expression of dominant-negative secreted VEGFR2/KDR and more importantly to inhibit the angiogenesis process [185].

A new generation of ASOs called TOES (Targeted Oligonucleotide Enhancers of Splicing) have been developed to induce the inclusion of otherwise skipped exons. TOES are complex modified antisense RNA oligonucleotides formed by two functionally distinct regions: the 5′ half of the oligo is complementary to a sequence within an exon of interest and is followed by a non-complementary RNA tail, designed to mimic an ESE sequence (Figure 1(c)). In this manner the oligo recruits specific *trans*-acting regulatory factors (such as SR proteins) and provides a sort of enhancer *in trans* that promotes exon inclusion [180, 186]. TOES have been first tested for their ability to induce the inclusion of *SMN2* exon 7 in spinal muscular atrophy (SMA) patient fibroblasts [187]. The TOES technology has been so far applied only once to correct splicing in cancer cells [176]. As described before, SRSF1 over-expression produces skipping of *Ron* exon 11 and the production of the oncogenic ΔRon isoform [176]. In order to correct pathological Δ*Ron* splicing, we have designed a TOES complementary to the first region of exon 11 and containing a tail of GGA repeats, known to function as a strong enhancer. This treatment was able to revert Δ*Ron* splicing and to increase exon 11 inclusion [176], suggesting the exciting possibility to consider splicing of exon 11 as a possible target of new anti-metastatic therapeutic approaches.

Another well-established method to down-regulate a specific splicing isoform is through RNA interference (RNAi). Small interfering RNAs (siRNA) are a class of double-stranded RNA molecules interfering with the expression of specific genes with complementary nucleotide sequence through endonucleases-mediated degradation mechanism [188]. Among the most recent studies on siRNA technology performed in xenograft tumor models, it is worth mentioning the article of Sangodkar and collaborators [130]. KLF6-SV1, an oncogenic splice variant of the tumor suppressor *KLF6* gene, is significantly up-regulated in several human cancers [81, 189] and its over-expression is associated with decreased survival in prostate and lung cancers [79, 190]. Sangodkar and colleagues demonstrated that knock-down of this variant via RNAi restored chemotherapy sensitivity and induction of apoptosis in lung cancer cells both *in vitro* and *in vivo* [130]. Conversely, over-expression of KLF6-SV1 resulted in a marked reduction in chemotherapy sensitivity in a tumor xenograft model.

Another target for siRNA-mediated anticancer therapy is hnRNP L. Like to SRSF1 [191], hnRNP L binds to a splicing regulatory element and regulates the splicing profile of *caspase-9* gene [129], which is altered in a large percentage of human lung cancer [192]. Recently, it has been reported that RNAi-mediated down-regulation of hnRNP L is sufficient to increase the caspase-9a/9b ratio and, more importantly, to cause a complete loss of tumorigenic capacity in xenograft model [192]. 

Finally, some of ASOs prevent ribosomal assembly and hence mRNA translation and seem to be well tolerated in patients [193]. An example of this approach is provided by survivin, an inhibitor of apoptotic proteins [194]. Survivin is expressed in several human cancers and its over-expression is associated with a poor prognosis [195]. These features make survivin an attractive target for anti-cancer therapy and several efforts, so far unsuccessful, have been made along this line. Recently, down-regulation of survivin has been achieved by using LY2181308, an ASO targeting the translation initiation codon of *survivin* mRNA and inducing its RNase H-mediated degradation [196]. LY2181308 treatment in multiple cancer cell lines caused apoptosis through activation of caspase-3. Most importantly, in a xenograft tumor model, LY2181308 produced significant anti-tumor activity and sensitized tumor cells to chemotherapeutic-induced apoptosis. All these findings led LY2181308 to be evaluated for clinical trial (Phase II) in combination with docetaxel for the treatment of prostate cancer, acute myeloid leukemia, and non-small cell lung cancer [196, 197].

## 5. Conclusions

The most important concept opened by the results reviewed here is that the RNA-binding proteins are at the centre of the oncogenic alternative splicing switch that controls all the major aspects of cancer cell biology (Figure 1). Understanding the molecular basis and the effects of the splicing regulation on the transcriptome of cancer cells promises to identify key circuits that have a fundamental role in cell proliferation, apoptosis, and other aspects of tumor progression. Moreover, despite the progress, significant challenges remain towards the rational design of more specific and selective approaches able to modulate alternative splicing events in order to control cancer growth.

In the era of the personalized medicine, each therapy would have to fit the combination of markers specific for each patient. Powerful and cost-effective methods are required to evaluate cancer markers, including those generated by alternative splicing, not only to provide a diagnosis and a prognosis but also to suggest the right personalized therapy.

## Figures and Tables

**Table 1:** Examples of genes that encode cancer-associated alternative splicing variants.

Gene	Splice variant	Cancer type	Function	Reference
*ABCC1 *	Various internal deletions	Ovarian cancer	Drug resistance	[127]
*Mdm2 *	Various internal deletions	Ovarian cancer	Loss of p53 binding	[127]
*Fibronectin *	Exclusion of EDB exon	Ovarian cancer	Tumor angiogenesis	[127]
*MENA *	Exclusion of exon 6	Breast cancer	Increase invasiveness and drug resistance	[104]
*MENA *	Skipping of exon 11A	Breast cancer	Enhance EMT	[103, 106]
*Ron *	Skipping of exon 11	Colon and gastric breast cancer	Increase motility and invasion	[64]
*HDMX *	Exclusion of exon 6	Soft-tissue sarcoma	Increase tumor aggressiveness	[132]
*p73 *	Inclusion of exon 13	Prostate cancer	Prostate hyperplasia and malignancy	[133]
*CASP9 *	Exclusion of a four-exon cassette	Non–small cell lung cancer	Susceptibility to chemotherapy	[134]
*CASP8 *	Retained intron	Breast cancer	Inhibition of caspase	[135]
*APC *	Skipping of exon 4	Colon cancer	Cause familial adenomatous polyposis (FAP)	[136]
*BRCA1 *	Skipping of exon 18	Breast cancer	Breast cancer susceptibility	[137]
*PTEN *	Retained introns 3 and 5	Breast cancer	Pathogenesis of sporadic breast cancers with p53	[137]
*p53 *	Retained intron	Breast cancer	Tumorigenesis	[137]
*KLF6 *	Alternative 5′ ss	Prostate cancer	Tumor cell proliferation	[82]
*NF1 *	Inclusion of exon 23a	Neurofibromatosis type I	Inactive tumor suppressor	[138]
*ASIP *	Alternative 3′ ss	Hepatocellular carcinoma	Blocks Fas-mediated apoptosis	[139]
*Bcl-X *	Alternative 5′ ss	Hepatocellular carcinoma	Regulation of apoptosis	[140]
*TACC1 *	Exon inclusion	Gastric cancer	Altered centrosome functions	[141]
*TERT *	Alternative 3′ ss	Astrocytic gliomas	Rescue of telomerase activity	[142]
*CDH17 *	Exclusion of exon 13	Hepatocellular carcinoma	Incidence of tumor recurrence	[143]

**Table 2:** Examples of ASO treatments in cancer cell lines.

Gene	Function	Reference
*SRA1*, Steroid Receptor RNA Activator gene	Retention of intron 1 alters the reading frame and occurs in breast tumors with high progesterone receptor contents	[144]
*Mcl-1*, myeloid cell leukemia-1	Antiapoptotic protein of the Bcl-2 family overexpressed in many tumors	[145]
*erbB-2*, Her-2 receptor	Skipping of exon 19 produces a dominant-negative protein isoform	[146]
*IG20*, death-domain adaptor protein Insulinoma-Glucagonoma 20	Antiapoptotic alternative splicing isoform expressed in gliomas	[147]
*CASP9*, caspase9	Two isoforms generated by alternative splicing: a proapoptotic and a prosurvival variant	[148]
*PKM*, pyruvate kinase M	The PKM2 isoform is crucial for aerobic glycolysis (the Warburg effect) and tumor growth	[149, 150]
*hTERT*, telomerase	Alternative splicing generates many nonfunctional products. ASOs treatment increases nonfunctional telomerase products in cancer cells	[151]
